# Gomesin inhibits melanoma growth by manipulating key signaling cascades that control cell death and proliferation

**DOI:** 10.1038/s41598-018-29826-4

**Published:** 2018-08-01

**Authors:** Maria P. Ikonomopoulou, Manuel A. Fernandez-Rojo, Sandy S. Pineda, Pablo Cabezas-Sainz, Brit Winnen, Rodrigo A. V. Morales, Andreas Brust, Laura Sánchez, Paul F. Alewood, Grant A. Ramm, John J. Miles, Glenn F. King

**Affiliations:** 1 QIMR, Berghofer Medical Research Institute, Herston, QLD 4002 Australia; 2 School of Medicine, The University of Queensland, Herston, QLD 4006 Australia; 3 Madrid Institute for Advanced Studies (IMDEA) in Food, CEI UAM + CSIC, Madrid, E28049 Spain; 4 Division of Chemistry and Structural Biology, Institute for Molecular Bioscience, The University of Queensland, St Lucia, QLD 4072 Australia; 5 Garvan Institute of Medical Research, Sydney, NSW 2010 Australia; 6 St Vincent’s Clinical School, University of New South Wales, Sydney, NSW 2052 Australia; 7 Departament of Zoology, Genetics and Physical Anthropology, University of Santiago de Compostela, 27002 Lugo, Spain; 8 Department of Medicinal Chemistry, Faculty of Pharmacy and Pharmaceutical Sciences, Monash University, Victoria, 3800 Australia; 9 Centre for Biodiscovery and Molecular Development of Therapeutics, AITHM, James Cook University, Cairns, Queensland Australia

## Abstract

Consistent with their diverse pharmacology, peptides derived from venomous animals have been developed as drugs to treat disorders as diverse as hypertension, diabetes and chronic pain. Melanoma has a poor prognosis due in part to its metastatic capacity, warranting further development of novel targeted therapies. This prompted us to examine the anti-melanoma activity of the spider peptides gomesin (AgGom) and a gomesin-like homolog (HiGom). AgGom and HiGom dose-dependently reduced the viability and proliferation of melanoma cells whereas it had no deleterious effects on non-transformed neonatal foreskin fibroblasts. Concordantly, gomesin-treated melanoma cells showed a reduced G0/G1 cell population. AgGom and HiGom compromised proliferation of melanoma cells via activation of the p53/p21 cell cycle check-point axis and the Hippo signaling cascade, together with attenuation of the MAP kinase pathway. We show that both gomesin peptides exhibit antitumoral activity in melanoma AVATAR-zebrafish xenograft tumors and that HiGom also reduces tumour progression in a melanoma xenograft mouse model. Taken together, our data highlight the potential of gomesin for development as a novel melanoma-targeted therapy.

## Introduction

Arthropods are the most abundant and widely distributed group of animals on earth. Within this group, spiders are one of the most speciose taxa, with over 47,000 species described to date^1^. Over a period of more than 400 million years^2^, spiders have evolved a myriad of venom peptides that are used for prey capture and/or defense against predators, as well as hemocyte-derived host-defense peptides that play a key role in innate immunity^3^.

According to the ArachnoServer database^4^, more than 40 antimicrobial peptides have been isolated from spider venoms. Despite their sequence diversity, all of these peptides are small (1.9–8.6 kDa) and highly cationic (pI 9.7–11.8). Moreover, in striking contrast with venom-derived peptide neurotoxins, all but four of these antimicrobial peptides are devoid of disulfide bonds. They are typically amphipathic and broadly cytolytic. They appear to be structurally disordered in aqueous solution but adopt an α-helical conformation in the presence of phospholipid membranes^5^. From an evolutionary perspective, it is striking that the vast majority of these antimicrobial peptides (39 in total) were isolated from the venoms of araneomorph (“modern”) spiders. The three exceptions are disulfide-rich neurotoxic peptides isolated from venom of the Chilean rose tarantula *Grammostola rosea*, a mygalomorph (“primitive”) spider. However, the antimicrobial activity of these peptides is likely to be secondary to their neurotoxic function.

Gomesin (AgGom), an antimicrobial peptide isolated from hemocytes of the South American mygalomorph spider *Acanthoscurria gomesiana*^6^, provides a striking contrast to the majority of antimicrobial peptides described to date; although small (18 residues) and highly cationic (pI 12.6, net charge + 6), gomesin it is not significantly amphipathic and it forms a well-defined tertiary structure comprising a β-hairpin that is stapled at each end by a single disulfide bond^7^. Moreover, both its N- and C-termini are protected by posttranslational modification (N-terminal pyroglutamate and C-terminal amidation). AgGom shares sequence and structural homology with the antimicrobial tachyplesin and polyphemusin peptides found in the hemocytes of horsehoe crabs^6,8^. Thus, AgGom belongs to an ancient and widespread family of invertebrate host-defense molecules. Hemocytes secreting AgGom have been shown to play a role in host defence against microbes^3^.

A myriad of activities have been reported for AgGom, including antibacterial, antifungal, and anthelminthic activities^6,8–10^. Although it possesses broad antimicrobial activity, AgGom is not as potent as the related tachyplesins and polyhemusins^8^. However, in contrast with most antimicrobial peptides, AgGom is not hemolytic and it is only weakly cytotoxic to HepG2 and HEK293 cells^8^. Perhaps more interesting from a therapeutic perspective is the report that AgGom is cytotoxic to a range of murine and human tumor cell lines, with topical gomesin shown to delay development of subcutaneous murine melanomas^11^. However, it remains to be determined how AgGom affects the molecular pathways driving cell proliferation and growth of melanomas.

Melanoma is an aggressive form of skin cancer with heterogeneous aetiology. It generates significant morbidity in part due to its high rate for developing chemoresistance^12^. In the US according to Skin Cancer Foundation statistics, one in every five Americans will develop skin cancer in their lifetime. Australia and New Zealand have the highest rates of melanoma in the world with an estimated 71 cases per 100,000 people, which translates to ~13,000 new cases every year^13^. Approximately 40–60% of all cutaneous melanomas carry mutations in the *BRAF* gene that cause constitutive activation of downstream mitogen-activated protein kinase (MAPK) signalling^14^. Approximately 90% of mutations in the *BRAF* gene result in the substitution of Glu for Val at codon 600 (*BRAF*^V600E^)^14^. *BRAF* encodes a RAS-regulated kinase that mediates cell growth and malignant transformation, and thus it is a promising drug target for treatment of melanoma^15^.

In this study, we investigated the anticancer properties of AgGom and a gomesin homolog (HiGom) in the melanoma cell line MM96L that contains the *BRAF*^V600E^ mutation. We shed light on the anticancer potency of these naturally occurring antimicrobial peptides and evaluate their functionality via a melanoma preclinical drug discovery platform. We show that gomesin peptides activate the Hippo signalling cascade and increase the cellular levels of the p53 and p21 cell cycle check-point proteins, whereas the MAPK pathway remains inhibited. Interestingly, gomesin peptides also activate the mTORC1, mTORC2 and AKT signalling pathways. We hypothesise that this activation occurs as a compensatory mechanism to prevent gomesin-induced apoptosis of MM96L cells. We determined that gomesin peptides reduce viability in a panel of BRAF-mutated melanoma cell lines and demonstrated that HiGom has more profound antiproliferative properties than AgGom in the tested melanoma lines. Both gomesin peptides abolished cell viability in two BRAF wild-type cell lines. Finally, we utilised two unrelated but complementary animal models namely the AVATAR-zebrafish and mouse xenograft models of melanoma, to study the antitumoral effect of gomesin peptides. Both peptides reduced tumor progression in the AVATAR-zebrafish xenograft model while in the mouse xenograft melanoma model we tested only HiGom (as a more powerful analogue) and showed that it limits tumour progression.

## Results

### Identification of venom-gland transcripts encoding gomesin

**Figure 1:**
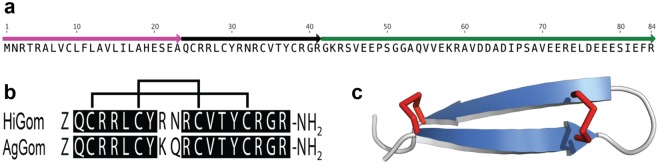
(**a**) Schematic of 84-residue precursor encoding the gomesin homolog HiGom. The signal peptide, mature gomesin, and propeptide are shown in magenta, black and green, respectively. Note that Z = pyroglutamate. (**b**) Sequence alignment showing amino acid identities (boxed in black) between HiGom and AgGom. Disulfide bond connectivities are shown above the alignment. **(c)** Schematic of the AgGom structure showing the disulfide-stabilized β-hairpin (PDB file 1KFP).

Sequencing and analysis of a venom-gland transcriptome from the Australian funnel-web spider *Hadronyche infensa* resulted in identification of numerous transcripts encoding toxins, putative toxins and proteins, most of which are likely associated with prey capture and defense. Amongst these transcripts, a cluster with seven reads was found to encode an ortholog (herein, HiGom) with sequence homology to that of the antimicrobial peptide gomesin (AgGom) isolated from hemocytes of the unrelated mygalomorph spider *Acanthoscurria gomesiana* (Fig. 1a).

The HiGom transcript encodes an 84-residue prepropeptide precursor comprised of a 23-residue signal peptide that precedes a single copy of the mature 18-residue HiGom peptide followed by a large propeptide region (Fig. 1a). The mature HiGom peptide contains an N-terminal Gln residue that we presume is post-translationally modified to pyroGlu as in the case of AgGom^6^. In addition, the propeptide region of the HiGom precursor contains a “KR” amidation signal immediately downstream of the final Arg residue in the mature toxin, and thus we predict that HiGom is C-terminally amidated like AgGom. The four-cysteine residues that form the two-disulfide bonds in AgGom are conserved in HiGom and homology modelling confirms that HiGom adopts the same disulfide-stapled β-hairpin structure as AgGom (Fig. 1b,c). We were unable to detect HiGom in milked venom, consistent with the low abundance of HiGom transcripts. However, although we did not recover any hemocycte-specific transcripts in the venom-gland transcriptome, we cannot exclude the possibility that the HiGom transcripts we identified arose from a small number of contaminating hemocytes in the venom gland preparation.

### Antimicrobial and hemolytic activity of AgGom and HiGom

AgGom and HiGom were chemically synthesized, oxidized to form the two disulfide bonds, and purified to >98% homogeneity using reverse-phase HPLC. To demonstrate functional homology between HiGom and AgGom, we tested the antimicrobial activity of both peptides against a variety of Gram-positive and Gram-negative bacteria (Table 1). Both AgGom and HiGom were active against Gram-positive and Gram-negative bacteria and in all cases HiGom was either equipotent with, or more active than, AgGom (Table 1). Both peptides were highly active against the soil firmicutes *Bacillus subtilis* and *Bacillus megaterium*. Neither peptide was active against *Streptococcus pneuomoniae* or methicillin-resistant strains of *Staphylococcus aureus*. In addition, both gomesin peptides showed minimal hemolytic activity even at doses as high as 460 μg/mL (Supplementary Fig. 1). In comparison, melittin, a well-studied cytolytic toxin from bee venom^16^ is hemolytic at much lower concentrations (2–9 μg/mL; see Supplementary Fig. 1).

**Table 1:** Antimicrobial activity of gomesin peptides from *A. gomesiana* (AgGom) and *H. infensa* (HiGom) against a range of Gram-positive and Gram-negative bacteria.

	MIC (μM)		MBC (μM)	
	AgGom	HiGom	AgGom	HiGom
**Gram-positive bacteria**				
*Staphylococcus aureus* ATCC 25923	>28	28	>28	28
*Enterococcus faecalis* ATCC 51299 VanB	28	14	28	14
*Enterococcus faecalis* Van A	7	3.5	7	7
*Bacillus subtilis* ATCC 6633	0.875	0.656	0.875	0.875
*Bacillus megaterium* ATCC 13632	≤0.014	≤0.014	≤0.014	≤0.014
*Streptococcus pneumoniae* ATCC 33400	>28	>28	>28	>28
*Staphylococcus aureus* ATCC 43300 MRSA	>28	>28	>28	>28
*Staphylococcus aureus* ATCC 29213 MRSA	>28	>28	>28	>28
*Staphylococcus epidermidis* GISA	14	7	14	7
**Gram-negative bacteria**				
*Escherichia coli* ATCC 25922	14	14	14	14
*Klebsiella pneumonia* ATCC 700603	>28	28	>28	28
*Klebsiella pneumoniae* ATCC 13883	14	7	14	7
*Pseudomonas aeruginosa* ATCC 10145	>28	>28	>28	>28

MIC = minimal inhibitory concentration. MBC = minimal bactericidal concentration. Three independent experiments were performed, each in triplicates.

### Gomesin peptides compromise the viability of BRAF mutated-melanoma cells

AgGom binds to cells with negatively charged outer membranes, including human MM96L melanoma cells, and forms pores in the membranes that decrease cell viability and proliferation^17^. To investigate whether HiGom shares similar cytotoxic properties, AgGom and HiGom were tested in melanoma BRAF-mutated MM96L cells and a non-transformed human neonatal foreskin fibroblast (NFF) cell line.

**Figure 2:**
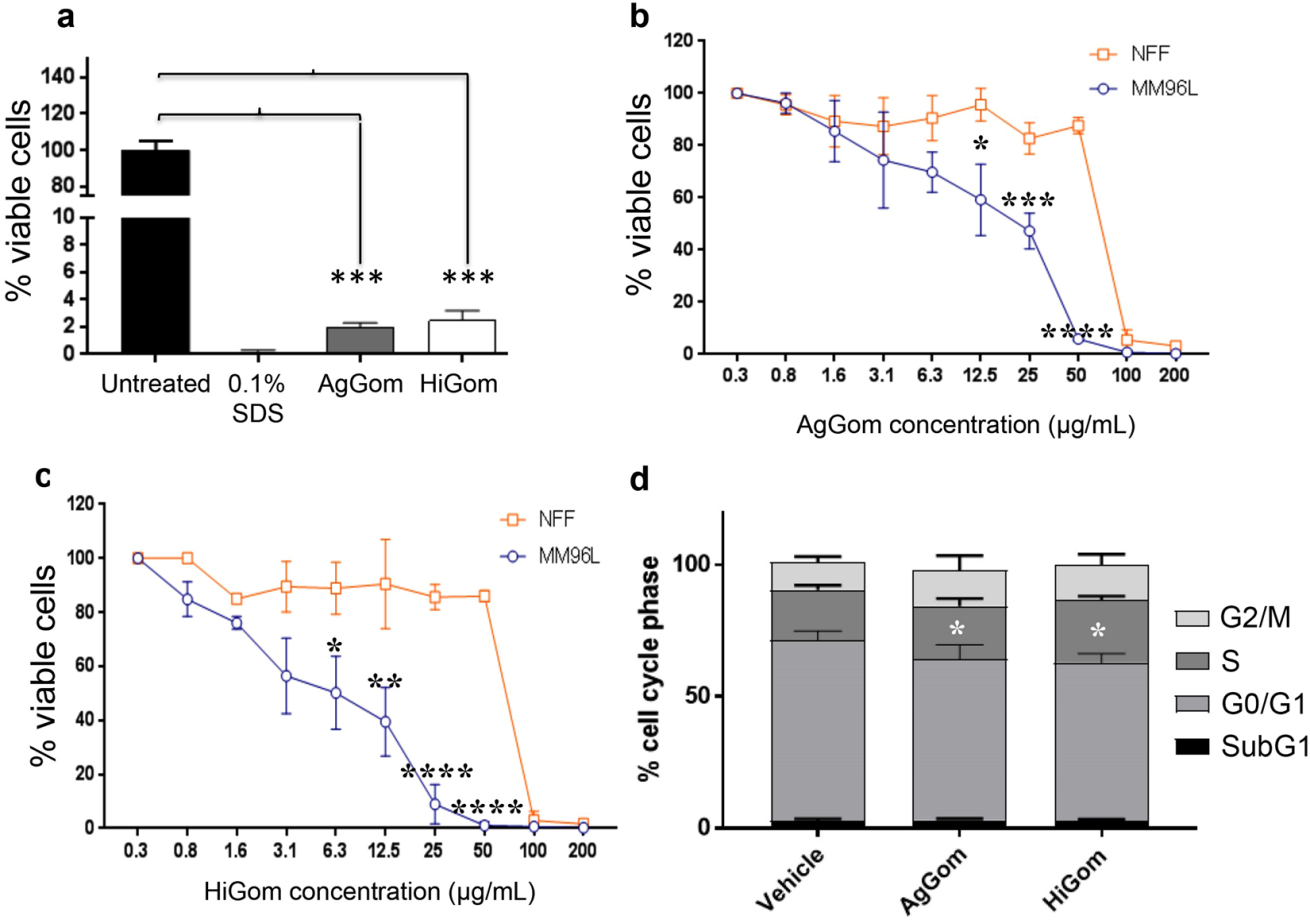
Gomesin peptides dramatically reduce viability of MM96L and BRAF-wild type cells. (**a**) Viability of MM96L cells treated with 100 µg/mL of AgGom or HiGom for 48 h, compared to cells treated with 0.1% SDS and untreated cells (**b**,**c**). Dose-response data for treatment of MM96L and NFF cells for 48 h with (**b**) AgGom or (**c**) HiGom. (**d**) Effect of gomesin peptides on cell cycle progression. MM96L cells were treated with 50 μg/mL AgGom or HiGom for 24 h. Changes in cell cycle (SubG0, G0/G1, S and G2/M phases) were analysed using the FloJow 10.06 program. Data are shown as mean ± SEM. Experiments were performed in triplicate and are the result of three independent experiments. *P < 0.05, **P < 0.01, ***P < 0.001, ****P < 0.0001 (MM96L vs NFF).

Treatment of MM96L cells for 48 h with 100 µg/mL AgGom or HiGom markedly reduced cell viability to < 5% (Fig. 2a). Similarly, reduced viability was observed in a panel of melanoma BRAF-mutated cells (MM96L, A2058, HTT144, JA, SKMEL28 and A02) at 50 µg/mL, with a more profound effect observed for HiGom (Supplementary Fig. 2). Importantly, treatment of MM96L cells with 3–50 µg/mL of AgGom or HiGom reduced MM96L viability without affecting the viability of NFF cells (ANOVA; F_9,11_ = 44.71; P < 0.0001) (Fig. 2b,c). HiGom had a stronger cytotoxic effect on MM96L cells than AgGom (EC_50_: 5.17 vs. 13.59 µg/mL, respectively; compare Fig. 2b,c), causing a significant decrease in MM96L viability at concentrations as low as 6.3 µg/mL (ANOVA, Bonnferroni’s multiple comparison test, F_3,8_ = 10.5; P = 0.038; Fig. 2c). Interestingly, we found that AgGom and HiGom are also highly cytotoxic to two BRAF wild-type cells (C001 and C002), suggesting that these peptides might have anti-proliferative activities in a wide range of melanoma cell types (Supplementary Fig. 3a,b). Moreover, to further assess the potential deleterious effects of AgGom and HiGom on a healthy skin cell line, we tested them in human melanocytes. While both peptides were lethal at 50 µg/mL (Supplementary Fig. 3c), they showed no cytotoxic effects at a concentration of 12.5 µg/mL (Supplementary Fig. 3d), a dose that effectively reduced MM96L melanoma cell viability (Fig. 2b,c).

### Gomesin peptides stimulate production of reactive oxygen species (ROS) and reduces mitochondrial membrane potential, resulting in late cell apoptosis

FACS was used to examine cell cycle progression in MM96L cells after 24 h exposure to 50 µg/mL AgGom or HiGom (i.e., the highest concentration that is non-toxic to NFF; Fig. 2b,c). In comparison to vehicle-treated cells, AgGom and HiGom-treated MM96L cells were in G0/G1 phase (*t*-test: DF = 6, P = 0.0112; P = 0.0404, respectively) and a greater number were arrested in S-phase (*t*-test: AgGom: p = 0.035; HiGom: p = 0.57, respectively) (Fig. 2d); thus, both gomesin peptides reduce cell proliferation at G0/G1-phase while AgGom caused cell cycle arrest at S-phase.

**Figure 3:**
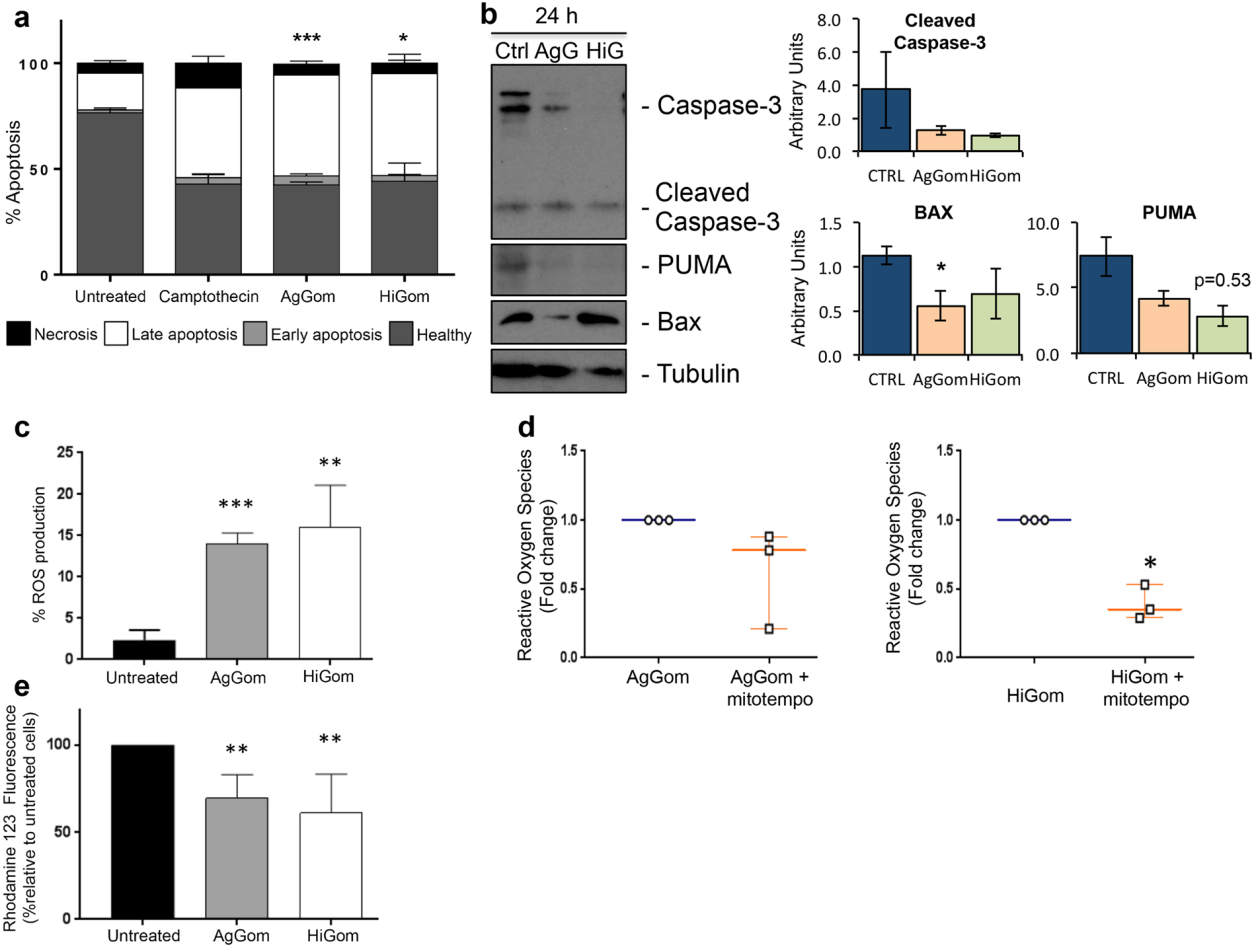
AgGom and HiGom induce late apoptosis of MM96L cells. (**a**) Apoptosis of MM96L cells incubated AgGom or HiGom (50 μg/mL for 48 h) was assessed using an Annexin V-FITC assay. Both peptides significantly increased the number of late apoptotic cells (>50%) in comparison to untreated cells. Cells treated with camptothecin (25 µM, 24 h) were used as a positive control. (**b**) Representative western blots and protein quantification from three independent experiments showing the pro-apoptotic cell markers cleaved Caspase-3, Puma, Bax and Tubulin in MM96L cells incubated with AgGom or HiGom (50 μg/mL, 24 h). (**c**) Gomesin peptides (50 μg/mL, 24 h) increased ROS production in MM96L cells as measured by flow cytometry. (**d**) MM96L cells were pre-treated for 2 h with mitoTEMPO prior to addition of 50 μg/mL AgGom or HiGom. Cells had reduced ROS production compared to cells treated with AgGom or HiGom alone. (**e**) The mitochondrial membrane potential of MM96L cells was reduced after treatment with AgGom or HiGom (50 μg/mL, 24 h). Data are shown as mean ± SEM and are the result of a minimum of three independent experiments performed in triplicate or duplicate (cell cycle experiments). Statistical analyses are relative to untreated cells and are represented as: *P < 0.05, **P < 0.01, ***P < 0.001.

Treatment of MM96L cells with 50 µg/mL AgGom or HiGom increased the number of late apoptotic cells by 2.7-fold compared to untreated cells (ANOVA, F_3,15_ = 7.393, P = 0.0029;) (i.e., AgGom: 47.7 ± 2.3, HiGom: 48.18 ± 9.1; Untreated: 17.4 ± 1.7; Fig. 3a). A similar result was seen using camptothecin, a well-known cytotoxic natural compound that induces apoptosis in cancer cells^18^ (Fig. 3a). The number of healthy cells decreased by 1.8- and 1.7-fold in AgGom- and HiGom-treated MM96L, respectively, compared to vehicle-treated cells (AgGom: 42.5 ± 1.4; HiGom: 38.85 ± 8.8; Untreated: 76.6 ± 2.4; ANOVA, F_3,15_ = 9.701, P = 0.0008) (Fig. 3a). However, no significant stimulation of early apoptosis or necrosis was observed in response to either gomesin peptide. Accordingly, analysis of pro-apoptotic markers at early stages of gomesin treatment (24 h, 50 µg/mL) did not reveal an increase the levels of cleaved PARP and caspase 3 or of Bax and Puma (Fig. 3b), implying that either apoptosis was not initiated at 24 h or that apoptosis is independent of the caspase 3 pathway. Interestingly, while the ROS output in vehicle-treated cells was minimal, MM96L cells exposed to AgGom or HiGom for 24 h generated a significant amount of ROS that may explain their apoptotic profile after 48 h (ANOVA, F_3,10_ = 8. 584, P = 0.0041) (Fig. 3c). Pre-incubation of MM96L cells with mitoTEMPO, a specific inhibitor of mitochondrial ROS production, prior to gomesin treatment prevented ROS production, consistent with HiGom stimulating ROS generation (*t*-test: t = 8.459, df = 2, p = 0.037) (Fig. 3d). As expected, a significant decrease in Rhod-123 fluorescence confirmed that the elevated generation of ROS in response to gomesin peptides was accompanied by a significant loss of mitochondrial membrane potential integrity (Kolmogorov-Smirnov test, AgGom: P = 0.0079; HiGom: P = 0.008) (Fig. 3e).

### Gomesin peptides inhibit the MAPK pathway but stimulate the Hippo pathway and p53/p21 axis

**Figure 4:**
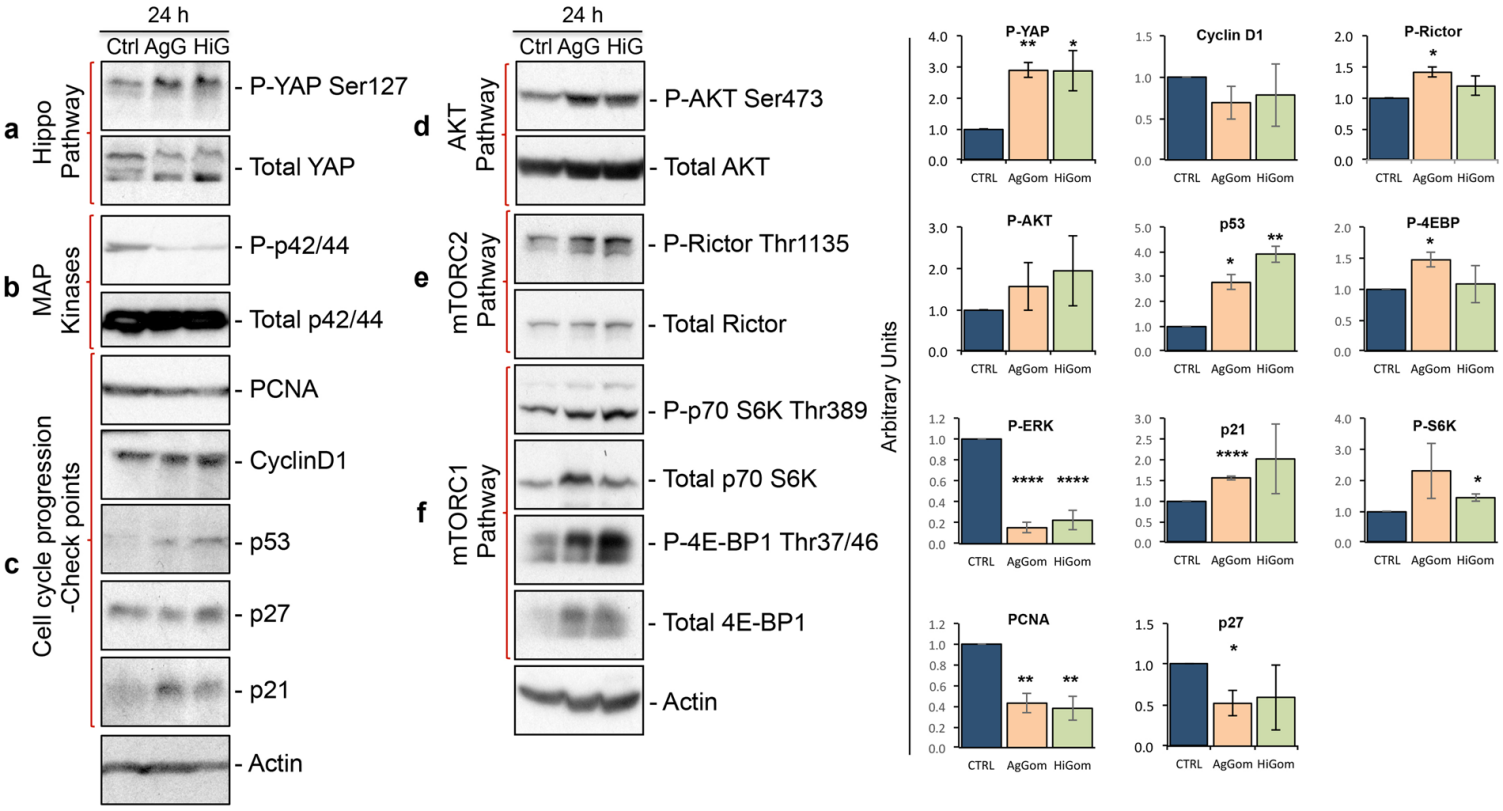
Gomesin peptides activate the Hippo pathway, inhibit MAPK cascades, and stimulate the p53/p21 cell cycle checkpoint axis. Representative western blots from three independent experiments showing: (**a**) Immunodetection of phospho-YAP^Ser127^ in comparison to total YAP protein as a marker of the Hippo pathway; (**b**) Phospho-p42/44 (also called Phospho ERK) as a marker of the level of activation of the MAPK pathway; (**c**) PCNA, cyclin D1, p53, p21 and p27 as markers of the checkpoints that regulate progression of the proliferative cell cycle. Actin was used as a protein-loading marker; (**d**) Phospho-AKT^Ser473^ in comparison to total AKT protein; (**e**) Phospho-Rictor^Thr1135^ in comparison to total Rictor protein as a marker of the activation of the mTORC2 complex/cascade which is responsible for phosphorylation of AKT at Ser473; (**f**) Phospho-p70S6K^Thr389^ in comparison to total p70S6K protein and Phospho-4E-BP1^Thr37/46^ in comparison to total 4E-BP1 protein as markers of activation of the mTORC1 complex/cascade which is responsible for the phosphorylation of Rictor at Thr1135. MM96L cells were treated with 50 μg/ml AgGom (AgG) or HiGom (HiG) for 24 h. Right panel shows quantification of the western blots.

We examined the molecular signaling cascades underlying the anti-proliferative and pro-apoptotic activities of AgGom- and HiGom-treated MM96L. We focused our investigations on the Hippo, AKT, mTOR and MAPK signalling pathways that have been described to mediate proliferation in melanoma cells^19–21^. The Hippo pathway regulates cell proliferation and controls organ size and growth by cytosolic sequestration and inhibition of the pro-proliferative activity of Yes-associated protein (YAP), via phosphorylation of YAP at Ser127 (P-YAP^Ser127^). Deregulation of the Hippo pathway and subsequent translocation of dephosphorylated YAP into the nucleus has been linked to the development of several types of cancer^21^. Examination of this pathway in cultured MM96L cells revealed elevated levels of P-YAP^Ser127^ in the presence of AgGom and HiGom (Fig. 4a), an indication that gomesin peptides activate the Hippo pathway to retain YAP in the cytosol and thus prevent its pro-proliferation activities.

In addition, AgGom and HiGom almost abolished phosphorylation of the p42/44 protein, suggesting that these peptides inhibit the MAPK pathway (Fig. 4b), a well-known molecular pathway that drives cell proliferation in cancer cells^22^. Subsequent analysis of the check-points responsible for normal cell cycle progression revealed elevated levels of p53 and p21 but not p27 in AgGom- and HiGom-treated MM96L cells (Fig. 4c). Gomesin peptides did not affect CyclinD1, a G1-phase marker. However, cell cycle arrest correlated with mildly decreased levels of the proliferating cell nuclear antigen (PCNA) (Fig. 4d), a marker of the progression of cells into S-Phase.

### Activation of pro-survival AKT and mTOR signaling in response to gomesin peptides

Paradoxically, we found that AgGom- and HiGom-treated cells exhibited over-phosphorylation of AKT at Ser473 (AKT^Ser473^) (Fig. 4e). While AKT phosphorylation and activation contribute to cell proliferation, they also constitute an anti-apoptotic molecular mechanism^23^. Indeed, it has been postulated that the primary function of the major AKT isoform, AKT1, is to promote cell survival. Since we demonstrated that AgGom and HiGom arrest melanoma cell proliferation, we suggest that activation of AKT signaling might be a compensatory cell survival mechanism to prevent apoptosis. Thus, we examined the molecular circuits underlying activation of AKT signaling for cell survival that comprises mTORC2- and mTORC1-target p70 S6Kinase. mTORC2 fully activates AKT by mediating phosphorylation at AKT^Ser473^ ^24^. In AgGom- and HiGom-treated MM96L cells we observed increased phosphorylation of Rictor at Thr1135 (Fig. 4f), implying mTORC2 activation^25,26^. The phosphorylation of Rictor at Thr1135 is mediated by phosphorylated p70S6 kinase at Thr389 (P-p70S6K^Thr389^) (Fig. 4f), which depends on activity of the mTORC1 complex^25–27^. Accordingly, we observed an increase of P-p70S6K^Thr389^ as well as an elevated phosphorylation of the mTORC1-target 4E-BP1 at Thr37/46 (P-4E-BP1^Thr37/46^) (Fig. 4g).

### HiGom delays progressive growth of xenograft melanoma tumours

**Figure 5:**
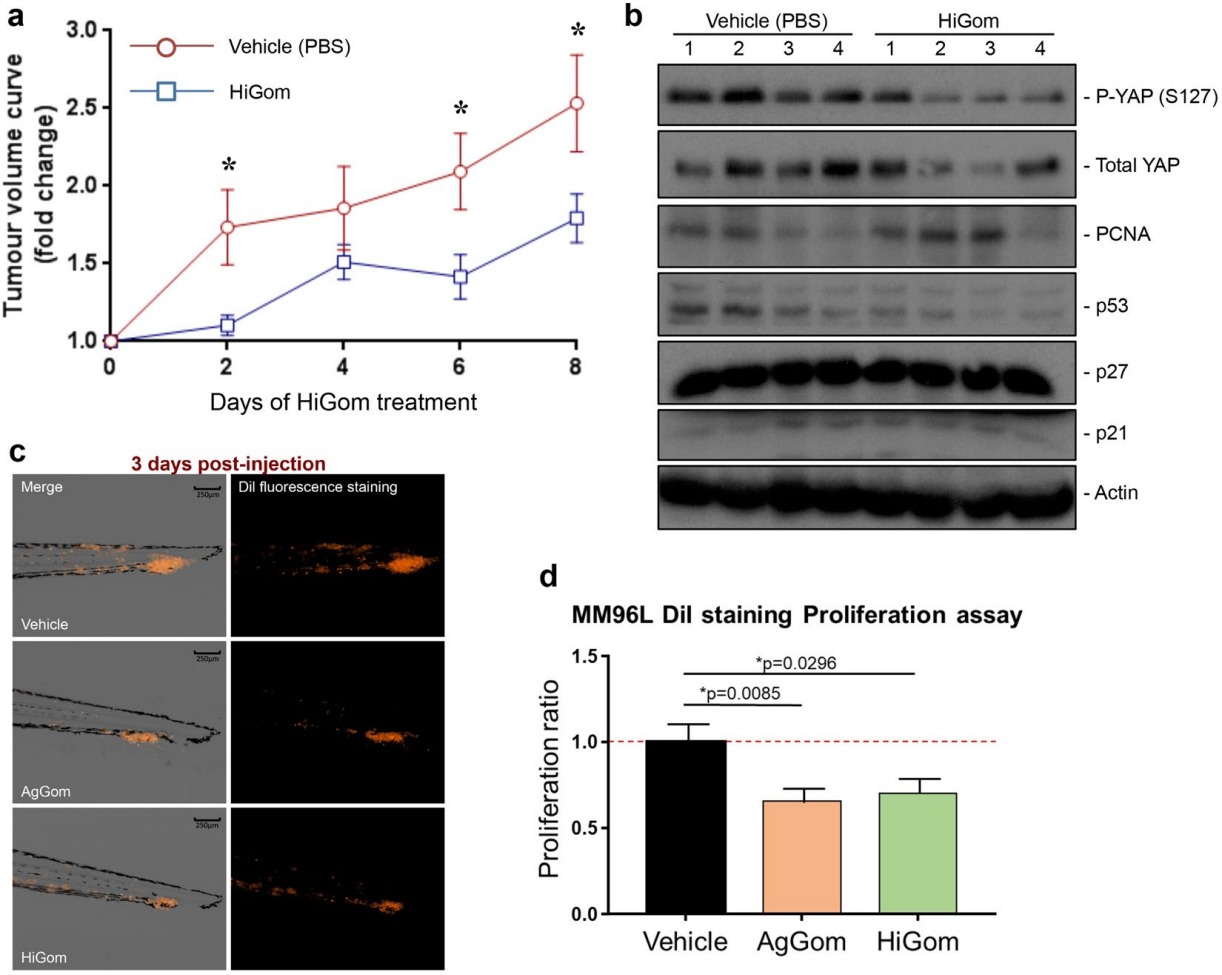
HiGom inhibits progression of xenografted human melanomas. (**a**) Nude mice bearing MM96L tumors were injected i.p. with 10 mg/kg HiGom or PBS (vehicle control group) at two-day intervals for a total of eight days, with tumour volume measured every two days and at the conclusion of treatment by digital callipers. Tumour progression was evaluated by assessing the fold-change in tumor volume every two days in comparison to the tumor volume observed prior to initiation of HiGom treatment. (**b**) Analysis of the signaling cascade in HiGom-treated in comparison to vehicle (PBS) treated- MM96L xenograft tumors. Ten mouse tumours per group were assessed. (**c**) Representative images of MM96L cells labeled with dil after 1-day post injection (1 dpi) and 3 dpi in zebrafish embryos treated with AgGom or HiGom at 0.1 μg/mL in comparison to the control (salt dechlorinated tap water exposed) embryos. The images are shown as contrast (grey scale) and fluorescent (black background) phase. (**d**) The proliferation ratio of MM96L cells treated with AgGom or HiGom at 0.1 μg/mL and the control (untreated) cells in AVATAR zebrafish models. All cells were labeled with dil staining. Data for mouse and zebrafish experiments are shown as mean ± SEM. *P < 0.05.

In order to evaluate the potential of gomesin peptides for development as therapeutic leads against melanoma we examined the ability of HiGom to limit progression of MM96L-melanoma tumour xenografts in immunodeficient nude mice. We chose HiGom over AgGom at 10 μg/kg for these studies based on its higher potency in melanoma cells and less deleterious effects on NFF and melanocytes cells *in vitro* (see Fig. 2b,c & Supplementary Fig. 2). Tumour progression was evaluated by assessing the fold-change in tumour volume every two days in comparison to the volume prior to initiation of HiGom treatment. We observed a significant delay in tumour growth in HiGom-treated versus PBS-treated mice (*n* = 10 tumours per group; permutation test, *p < 0.05) (Fig. 5a). In contrast to cultured MM96L cells, we did not observe activation of the Hippo pathway or of the cell cycle markers PCNA, p53, p27 and p21 in the xenografted MM96L tumors of HiGom-treated mice compared to the vehicle-treated group (Fig. 5b).

We also exmained the anti-tumoral effects of AgGom and Higom in the AVATAR MM96L-xenograft tumor zebrafish model. Xenografted zebrafish embryos were intravenously injected with MM96L dil labeled cells to assess the efficacy of AgGom and HiGom on the proliferation and metastatic capacity of melanoma cells. At one-day post injection (dpi) the embryos were imaged and treated with 0.1 µg/mL of AgGom or HiGom, the maximum safe dose for the embryos (Supplementary Table 1). At 3 dpi the embryos were photographed again to allow comparison with images taken at 1 dpi in terms of total fluorescence (number of pixels in the image and the intensity of the fluorescence) and to measure the proliferation of the MM96L cells after being treated with AgGom or HiGom. Both AgGom and HiGom yielded a significant reduction (35% and 30%, respectively) in the proliferation of MM96L cells (*p < 0.05) compared to control cells, which translated into a significantly reduced tumor growth and invasive capacity of MM96L cells (Fig. 5c,d).

## Discussion

### Gomesin kills melanoma cells by induction of late apoptosis

Our investigations revealed that both AgGom and HiGom reduce the viability of melanoma cells at doses that are innocuous to non-transformed fibroblasts. HiGom exhibited greater anti-proliferative activity against a range of BRAF-mutated melanoma cell lines *in vitro* as well as lower cytotoxicity to NFF cells, making it a better candidate for development of an anti-melanoma drug. The different effect of AgGom and HiGom could be explained due to amplification of microphthalmia-associated transcription factor (MITF) expression in the different melanoma lines, which has been linked to MAPK inhibition^28^. Melanoma-bearing mice treated for eight days with HiGom showed significantly slower tumour progression in comparison to vehicle-treated controls. Consistent with this observation, it was previously shown that AgGom incorporated into a topical cream applied for approximately four weeks to C57BL/6 mice bearing B16F10-Nex2 subcutaneous tumours caused a significant delay in tumor progression^11^. However, the current study is the first to show that gomesin peptides inhibit growth of human melanoma tumors *in vivo*. In addition, independent and unbiased validation of our mouse results in an AVATAR MM96L xenograft tumor model in zebrafish^29^ highlights the therapeutic potential of gomesin against human melanoma BRAF-mutated cells.

AgGom and HiGom induce late apoptosis of melanoma cells. Apoptosis can be triggered as a cellular response to oxidative stress, caused by an imbalance between excessive ROS production and the response of the antioxidative defence mechanisms of cells^30^. While ROS output in vehicle-treated MM96L cells was minimal, a 24 h exposure to either AgGom or HiGom significantly elevated ROS production, which may in part explain the apoptotic effects of these peptides. Previous reports have linked increased intracellular ROS production and mitochondrial malfunction leading to apoptosis in AgGom-treated CHO cells^31^, as well as loss of mitochondrial membrane potential and cellular apoptosis related to the mitochondrial pathway in melanoma cells^32^. We found that that gomesin-treated MM96L cells had reduced mitochondrial membrane potential, and that gomesin-induced ROS production could be reversed by pre-treatment with mitoTEMPO. Taken together, our data suggest that gomesin peptides might impair mitochondrial function resulting in cell apoptosis.

### Mechanism of gomesin-induced death of melanoma cells

This is the first study to provide mechanistic details of how the arachnid-derived gomesin peptides, initially characterized as having antimicrobial, antifungal and anthelmintic activities, exhibit antiproliferative properties against human melanoma. We studied signaling cascades known to drive proliferation of melanoma cells, including the Hippo, AKT and MAPK pathways^19–21^.

The Hippo pathway mediates organ size and tissue growth by exerting strict control on the cellular proliferation rate. Consequently, Hippo pathway deregulation has been directly linked to the development of several types of cancer^33^. The transcription factor YAP is widely recognized as the central mediator of the pro-tumorigenic effects of Hippo pathway inactivation via its promotion of cell proliferation and inhibition of apoptosis^33^. To prevent the proliferative activity of YAP, the Hippo pathway via activation of the LATS1/2 kinases promotes phosphorylation of YAP at Ser127. This post-translational modification drives translocation of YAP from the nucleus to the cytoplasm, where it is sequestered through association with 14-3-3 proteins^33^. We showed that both AgGom and HiGom increase phosphorylation of YAP, thereby inhibiting the MAPK pathway^22^, a molecular pillar that sustains growth of human BRAF melanoma. We postulate that in order to inhibit proliferation and cell viability, and promote apoptosis in human BRAF MM96L cells, gomesin peptides coordinate activation of the Hippo pathway and inhibition of the MAPK signaling cascade.

AgGom and HiGom also enhance phosphorylation of AKT at Ser473 after 24 h, reflecting activation of the AKT pathway. The AKT signaling cascade is a pro-proliferative, anti-apoptotic pathway^23^. While activation of this pathway seems inconsistent with the pro-apoptotic profile of gomesin-treated MM96L cells observed at 48 h, we hypothesize that increased phosphorylation of AKT at Ser473 in response to 24 h of gomesin treatment might constitute a cell survival mechanism designed to delay apoptosis.

Cell survival and inhibition of pro-apoptotic signals is driven by P-p70S6K^Thr389^ (refs^34,35^) and mTORC2^36^. Our initial hypothesis postulated that the pro-survival activity of AKT might be the consequence of direct regulation of the mTORC1 target p70S6 kinase over the mTORC2 complex, which mediates phosphorylation at Ser473 and total activation of AKT. This hypothesis is supported by the fact that mTORC2 fully activates AKT by facilitating phosphorylation at AKT^Ser473^ (refs^25,26,37^). Accordingly, we showed that MM96L cells treated for 24 h with AgGom or HiGom increased the levels of phosphorylated p70S kinase, which consequently phosphorylates Rictor at Thr1135 (ref.^38^). However, the significance of phosphorylation of Rictor at Thr1135 is a subject of current controversy. Independent studies have postulated three different theories regarding phosphorylation of Rictor at Thr1135: an inhibiting^38^, activating^25^ or an indifferent^38^ post-translational event for the activity of AKT. Hence, it is difficult to assess the functional role of mTORC2 in MM96L cells in response to gomesin treatment. Moreover, it is possible that p70S6K^Thr389^ coordinates with AKT but in an AKT-independent manner^34,35^ to mediate pro-survival signalling in gomesin-treated cells. In addition, it has been shown that Hippo pathway activation and cytoplasmic retention of YAP is an AKT-dependent mechanism^33^. Therefore, it is also feasible that active AKT contributes to Hippo pathway function in order to mediate the anti-proliferative effects of gomesin. Clearly, further investigations are warranted into the role played by AKT in gomesin-induced inhibition of melanoma cell proliferation.

Previous investigations have suggested that the cytotoxicity of AgGom and its enhancement of ROS production in human neuroblastoma and rat pheochromocytoma cells is mediated via activation of the PI3K (which phosphorylates and contributes to the activation of AKT), MAPK and PKC signaling pathways^39^. These conclusions were based on data showing that inhibitors of MAPK, PKC and PI3K ameliorated AgGom-induced ROS production and release of lactate dehydrogenase. However, in contrast to our *in vitro* assays which employed MM96L cells cultured in medium containing fetal bovine serum (10%), serum was absent from the culture medium in the study by Soletti and colleagues^39^. Since the culture of cells in serum-free media arrests cell proliferation, some of the results observed by Soletti and colleagues could simply be off-target effects due to the lack of serum growth factors. Moreover, the lack of biochemical validation of these MAPK, PKC and PI3K inhibitors (*i.e*., examination of p42/44 and AKT phosphorylation as a readout of MAPK and PKC signaling) in AgGom-treated cells complicates interpretation of previous results. Nevertheless, it remains possible that AgGom engages different cell machinery in different tumors such as neuroblastoma and melanoma, which could explain the discrepancy between the two studies^39^.

Biochemical analysis of the signaling cascades controlling proliferation of MM96L indicate that AgGom and HiGom both arrest MM96L cells at S-phase and reduce the number of cells in G0/G1 phase. This is an intriguing and unusual phenotype since most cells are unable to enter S phase in response to cytotoxic agents. Interestingly, the cell-cycle phenotype of gomesin-treated MM96L cells mimics that induced by direct inhibitors of DNA replication such as hydroxyurea, fluorouracil and aphidicolin. In support of this analogy, analysis of the cell cycle checkpoints (*i.e*., p53, p21 and p27)^40^ and markers (cyclin D1 and PCNA)^22,40^ driving cell proliferation of MM96L cells, revealed that both gomesin peptides provoke cell cycle arrest through activation of the p53/p21 axis but not p27, which manifests as diminished expression of the S-phase marker PCNA^41^. p53 inhibits DNA replication^42^, while p21 mediates cell cycle arrest in G2 (G2 exit) and leads to senescence^43^, which might contribute to the late apoptosis observed in gomesin-treated cells. Intriguingly, cyclin D1 remained at constant levels. This could either be because cells could progress from G0 to G1 but not to S-phase, or because cyclin D1 does not play a role in cell cycle progression in MM96L cells, as seen in resistant metastatic melanoma lines^41^.

### Summary

This study is the first to demonstrate the potential of AgGom and HiGom in treating human melanoma containing BRAF mutations. We showed that gomesin peptides induce cell cycle arrest, reduce cell viability and stimulate apoptosis of MM96L cells. Mechanistically, our data revealed that gomesin exerts its anti-proliferative effect via three different molecular pathways: activation of the Hippo pathway, inhibition of MAPK cascades, and stimulation of the p53/p21 cell cycle checkpoint axis. In addition, activation of AKT might play a dual role by contributing to the Hippo pathway control of cell proliferation but as a survival signal that delays entry into apoptosis. Finally, our demonstration that gomesin inhibits the growth of human melanoma in two independent and unbiased *in vivo* melanoma xenograft tumor models suggests that it may have therapeutic utility.

## Methods

### Reagents

Fmoc–protected L-amino acids Arg(Tos), Asn(Trt), Cys(Trt), Cys(Acm), Gly, Leu, Lys(Boc), Thr, Tyr(tBu) and Val were purchased from Novabiochem (Sydney, Australia). Unprotected L-pyroglutamic acid, *triisopropylsilane* (TIPS), diethyl ether, iodine, ascorbic acid and ammonium bicarbonate were purchased from Sigma-Aldrich (Sydney, Australia). Fmoc Rink-amide resin was obtained from the Peptide Institute (Osaka, Japan). *O*-Benzotriazole-*N*,*N*,*N*′,*N*′-tetramethyl-uronium-hexafluorophosphate (HBTU) was from Iris Biotech (Marktredwitz Germany), while *N*,*N*-dimethylformamide (DMF), trifluoroacetic acid (TFA), piperidine, and *N*,*N*-diisopropylethylamine (DIEA) were from Auspep (Melbrourne, Australia). HPLC-grade acetonitrile was obtained from Merck (Merck, Pty, Australia), bacterial culture media from Bacto Laboratories (Australia), and enzymes (vancomycin and colistin) from Invitrogen and Life Technologies (Australia).

All media for cell culture was purchased from Invitrogen/Gibco. For melanocytes we used complete media acquired from ThermoFisher. MitoTEMPO was from Cayman Chemicals (#16621). The majority of antibodies were obtained from Cell Signaling Technology including Rabbit anti-Phospho AKT^Ser473^ (#4060), Total AKT (#4691), Phospho-YAP^Ser127^ (#13008), Total YAP (#8418), p21 (#2947), p27 (#3696), and p53 (#2527). Puma was purchased from ProSci (#3043), Bax was obtained from Merck (#ABC11), Cyclin D1 (#2978), PCNA (#13110), Phospho-p42/44 (#4370), Total ERK (#4067), Phospho-Rictor^Thr1135^ (#3806), Total Rictor (#2114), Phospho-p70 S6K^Thr389^ (#9234), Total p70 S6K (#2708), Phospho-4E-BP1^Thr37/46^ (#2855), Total 4E-BP1 (# 9644), Caspase cleaved 3 (#9929), PARP cleaved (#9929), and actin (#4970) were from Cell Signaling Technology. Anti-rabbit HRP was from Sigma (#A0545).

### Venom-gland transcriptome

Three days after milking to deplete their venom glands and stimulate toxin transcription, three spiders were anesthetized and their venom glands dissected out and immediately placed in TRIzol® reagent (Life Technologies). Total RNA was extracted and RNA quantity and integrity determined from the OD_260_/OD_280_ ratio and analysis on a Bioanalyzer 2100 Nano Chip (Agilent Technologies). mRNA enrichment from total RNA was performed using an Oligotex direct mRNA mini kit (Qiagen) following the manufacturer’s specifications. After elution, mRNA was precipitated with RNAse/DNAse-free ammonium acetate, glycogen and 95% ethanol. The resulting pellet was washed with 75% diethyl pyrocarbonate (DEPC)-treated ethanol and re-suspended in 25 μl of RNAse/DNAse-free water. Approximately 100 ng of mRNA was used to construct the cDNA library using the standard cDNA rapid library preparation and emPCR method recommended by Roche. After construction, libraries were sequenced by the Brisbane node of the Australian Genome Research Facility using a Roche GS FLX sequencer.

The Raw Standard Flowgram File (.SFF) was processed using Cangs software, and low quality sequences discarded using a Phred score cut-off of 25^44^. *De novo* assembly was done using MIRA software v3.2^45^ using the following parameters: -GE:not = 4–project = Hinfensa–job = denovo,est,accurate,454 454_SETTINGS -CL:qc = no -AS:mrpc = 1 -AL:mrs = 99,egp = 1. The assembled data set was visualized using TABLET or Geneious software^46,47^. An “in-house” algorithm was used to predict open reading frames encoding disulfide-rich toxins. Signal sequences were determined using the SignalP algorithm^48^. Putative propeptide cleavage sites were predicted using a sequence logo analysis of all known spider precursors. Homology of transcripts was determined via BLAST.

### Chemical synthesis of gomesin peptides

Gomesin peptides from *A. gomesiana* (AgGom) and *H. infensa* (HiGom) were synthesized manually at 0.2 mmol/g scale on a Rink amide linker loaded polystyrene resin using standard Fmoc solid-phase peptide synthesis (SPPS) protocols^49^. Selective disulfide formation was used to ensure that the native disulfide isomer of gomesin was obtained (i.e., CysI–CysIV, CysII–CysIII)^7^. To achieve selective disulfide pairing, Cys(Acm) was introduced in positions 6 and 11 whereas the cysteine residues in positions 2 and 15 were introduced as Cys(Trt). Briefly, at each cycle, the swollen resin was deprotected in 40% piperidine/DMF and coupled to the HBTU/DIAE-activated Fmoc amino acid for 20 min. At the end of the synthesis, the peptide chain was deprotected and cleaved from the protected peptide-resin using a solution of TFA:TIPS:water (90:5:5) for 3 h, then evaporated by a stream of N_2_. The crude peptide mixture was precipitated in ice cold diethyl ether and filtered. The crude peptide product was dissolved in a solution of 50% acetonitrile (ACN) acidified with 0.1% TFA, lyophilized, and purified using reversed-phase HPLC (RP-HPLC) (Supplementary Fig. 3a).

Folding via selective oxidation of each cysteine pair was achieved as shown in Supplementary Fig. 3a–c ^49,50^. The first disulfide bridge between Cys2 and Cys15 was formed by incubating the purified linear peptide in 100 mM ammonium bicarbonate (pH 8) at a final concentration of 0.1 mg/mL for 24 h. The partially folded peptide was lyophilized and resuspended in 50:50 acetonitrile:water solution acidified with 0.01% TFA. The Cys6–Cys11 disulfide bridge was then formed by dropwise addition of the peptide solution to an equal volume of 10 mM iodine dissolved in ACN/water/TFA (49:49:2). The reaction mixture was agitated for 20 min then quenched with ascorbic acid. The crude peptide was immediately purified using RP-HPLC. The formation of each disulfide bridge was monitored and confirmed using ES-LC-MS (ABI 150EX, AB-Sciex (See Supplementary Fig. 3b,c). Homogeneous peptide fractions were lyophilized and high-resolution mass data was obtained by LC-TOF-MS analysis (QSTAR Elite; AB-Sciex). AgGom had a monoisotopic mass of 2269.0734 (predicted = 2269.0717), while HiGom had a monoisotopic molecular mass of 2284.6972 (predicted = 2284.6950). Peptides were stored at −20 °C until further use.

### Antimicrobial assays

The antibacterial activity of AgGom and HiGom was measured using a microtiter broth dilution method^51^. Briefly, Gram-positive and Gram-negative bacteria were cultured in Muller Hinton II broth at 37 °C. Overnight bacterial cultures were diluted 40-fold into fresh broth and incubated at 37 °C for 2–3 h. The resultant mid-log phase cultures (OD_600_ ~0.6) were diluted to a final concentration of 5 × 10^5^ colony forming units (CFU)/mL and added to assay wells containing 2-fold serial dilutions of synthetic gomesin. Plates were incubated at 37 °C for 24 h, and the minimum inhibitory concentration (MIC) was taken as the lowest concentration of gomesin that yielded no visible bacterial growth. Vancomycin and colistin at a concentration of 1.28 mg/mL were used as positive controls. To determine the minimal bactericidal concentration (MBC), 30 µL of resazurin (0.01%) was added to each well after the MIC values were determined; then plates were further incubated at 37 °C for 24 h. Wells with blue or pink colouration indicated dead or live bacteria, respectively. The MBC value was determined as the lowest gomesin concentration that yielded blue colouration. Assays were performed in triplicate.

### Hemolytic assay

Hemolysis was monitored by adding synthetic gomesin (6.25–187 μM) to human erythrocytes (0.25% in phosphate buffered saline). 1% Triton-X100 was used as a positive control (100% lysis). Samples were incubated at 37 °C for 1 h, centrifuged at 400 *g* for 5 min, then the absorbance of the supernatant (100 μl) measured at 415 nm using a microtiter plate reader (BIOTEK PowerWave XS). Melittin (0–3.33 μM), a highly hemolytic bee-venom peptide, was used for comparison. Experiments were performed in triplicate.

### Cell culture

The human melanoma cell lines MM96L, A2058, HTT144, JA, SKMEL28, A02, C001, C002 as well as the nontransformed NFF cell line and melanocytes were maintained in a humidified incubator at 37°C and 5% CO_2_. All melanoma cells were cultured in RPMI-1640 media supplemented with 10% FCS,and 2 mM Glutamax^TM^. NFF cells were grown in RPMI-1640 containing 10% FCS and melanocytes were maintained in medium 254CF supplemented with Human Melanocyte Growth Supplement (HMGS). Penicillin/streptomycin (PS) (100 U/ml each) were added to all media. Cells were passaged at approximately 90% confluency. Functional studies were performed with passages up to 25. All cell lines were genotyped to ensure purity and absence of mycoplasma contamination.

### Cell viability assay

Cell viability was measured via a commercial colorimetric assay based on the reduction of the yellow tetrazolium dye MTT (Sigma-Aldrich) to purple formazan by NAD(P)H-dependent oxido-reductases in living cells. Thus, MTT reduction is a reflection of the number of viable and proliferative cells. We assessed cytotoxicity by comparing melanoma (MM96L, A2058, HTT144, JA, SKMEL28, A02, C001, C002), melanocytes and NFF cells. Briefly, 2,500 or 5000 melanoma and 8,000 or 4,000 (NFF & melanocytes) cells/well were seeded in a 96-flat microtiter well plate for 24 h to allow cell adhesion. Gomesin peptides were then added and MTT reduction measured after 48 h from the absorbance at 540 nm measured on a microplate reader (BIOTEK PowerWave XS). 0.1% SDS was used as a positive control (100% toxicity). The concentration of gomesin peptide causing 50% inhibition (IC_50_) was determined was determined using GraphPad Software, Inc (USA).

### Flow cytometry

MM96L cells were treated for 24 h with 50 µg/mL AgGom or HiGom, the maximum safe dose that killed MM96L cells but had minimal effect on NFF. Cells were then trypsinised, washed with PBS, and fixed with 70% ethanol for ~1 h at 4 °C. Ethanol was removed by centrifugation (453 *g* for 5 min), then cells were washed with PBS. Cells were then treated with 5 µl of 10 µg/ml ribonuclease A to remove contaminating RNA and were further incubated for 1 h at 37 °C to allow release of low-molecular weight DNA. Cell pellets were stained with 10 µl of propidium iodide (PI) (1 mg/ml stock) and analysed at a maximum emission of 605 nm using a BD LSR Fortessa 5 analyser (BD Biosciences). A minimum of 10,000 events were recorded. All data were analysed using FlowJo v10.06.

### Apoptosis assay

The level of apoptosis in MM96L cells treated with AgGom or HiGom, was measured by FACS using an Annexin V-FITC apoptosis detection kit (BD Biosciences). Briefly, cells were seeded at a density of ~100,000 in a round-bottom 96-well plate and treated with 50 µg/mL AgGom or HiGom for 48 h. Cells were then harvested with accutase, and washed twice with PBS and once with wash buffer provided by the manufacturer. Cells were stained simultaneously with FITC-labeled annexin V and PI. Stained cells were kept in the dark at room temperature and analysed using a BD FACS Canto™ II high-throughput system (BD Biosciences) within 30 min of staining.

### ROS assay

An Amplex® Red Hydrogen Peroxidase assay kit (Invitrogen) was used to determine whether AgGom or HiGom caused intracellular toxicity in MM96L cells by generation of ROS, a known marker of intracellular oxidative stress in response to environmental noxious conditions. Carboxy-H2DCFDA (Invitrogen), a fluorescence probe that detects intracellular hydrogen peroxide, was added 30 min prior to harvesting cells to measure ROS production in MM96L cells. Fluorescent cells were first washed twice with PBS before being analysis with a BD FACSCalibur™ flow cytometer (BD Biosciences) using excitation and emission wavelengths of 492 nm and 517 nm, respectively. Approximately 10,000 events were recorded per sample and the read-out was analysed using FlowJo v10.06. In a separate series of experiments, MM96L cells were pre-treated with 10 µM mitoTEMPO, a mitochondrially-targeted superoxide scavenger, for 2 h prior to gomesin addition.

### Mitochondrial membrane potential

Rhodamine 123 (Rhod-123) is a cationic dye that is sequestered by mitochondria and thus can used to assay mitochondrial membrane potential (MMP). Loss of MMP results in loss of Rhod-123 fluorescence. MM96L cells (~1 × 10^6^) were resuspended in 0.1 mL of culture medium, stained with 10 µg/m L Rhod-123 for 30 min, and then washed twice with PBS. The intracellular concentration of Rhod-123 was then determined immediately via flow cytometry using an excitation wavelength of 488 nm. Data were analysed using FlowJo v10.06.

### Western blots

MM96L cells were lysed in cold RIPA buffer containing protease (Merck Pty Ltd, Kilsyth, Australia) and phosphatase (Roche Diagnostics, Castle Hill, Australia) inhibitors and stored at −20 °C. Protein concentrations were determined using a Pierce BCA Protein assay kit (Thermo Fisher Scientific). Samples were subjected to SDS-PAGE and blotted according to standard procedures. In brief, 10 μg of protein was loaded per lane. Antibodies used for western blots are described in the Reagents section. Protein signals were visualized using enhanced chemiluminescence (Pierce™ ECL Western Blotting Substrate).

### Animal studies

All mouse work methods were carried out in accordance with the QIMR Berghofer Medical Research Institute relevant ethical guidelines and regulations. In addition, all experimental protocols were approved by the QIMR Berghofer Medical Research Institute Ethics and Animal Committees (Project P2109 & Ethics Permit A15605).

To determine the maximum safe dose of HiGom, two mice initially received an intraperitoneal (*i.p*.) injection of HiGom (12.5 µg/kg, diluted in PBS). Mice were closely monitored for two days to check for possible side effects. Every two days and for a total of three weeks thereafter the peptide dose was increased by 50%. The HiGom dose was safely incremented up to 15 mg/kg.

Ten female seven week old BALB/c FoxN1 nude mice were injected subcutaneously (*s.c*.) with 2.5 × 10^6^ MM96L cells in both flanks to develop two tumours per mouse. Two weeks later, when the tumours were of palpable size, we initiated treatment with HiGom (10 mg/kg). Briefly, mice were divided in two groups of five, which were treated with vehicle (PBS) or HiGom. Every two days and for a total of eight days, mice were injected *i.p*. with either HiGom or PBS in a final volume of 100 μl. To assess the impact of HiGom on tumour growth, the dimensions of the xenograft tumours were estimated using a digital calliper and applying the algorithm: *Volume = a x b x b x 0.5*, where “*a*” is the length and “b” is the measured breadth of the tumour lump (skin included)^52^. The effects of HiGom on tumour progression were estimated by calculating the fold-change in size versus the initial size prior to the first injection of HiGom (day 0). At the end of the experiment, mice were euthanised with CO_2_ and tumours were harvested for histology (Haemotoxylin and Eosin staining).

### Zebrafish handling and care

Zebrafish embryos were obtained by mating adult zebrafish (*Danio rerio*, wild-type) that were maintained in 30 L of aquaria at a ratio of one fish per liter of water with a light-dark cycle of 14:10 h and at a temperature of 28.5 °C according to previously described procedures^53^. All experimental procedures, fish care and treatments were performed with the approval of the Animal Care and Use Committee of the University of Santiago de Compostela and the standard protocols of Spain (Directive 2012-63-DaUE). At the end of the experiments, zebrafish embryos were euthanised by tricaine overdose.

### Zebrafish toxicity assays

A toxicity assay for AgGom and HiGom peptides was performed to assess the mortality of zebrafish embryos exposed to different peptide concentrations and at an incubation temperature of 28.5 °C. This assay was performed using 12 replicates per concentration and is the result of three different independent experiments. In brief, we exposed embryos at 72 h post fecundation (hpf) to the dissolved peptides for five days (until the embryos reached 192 hpf). The concentrations used were 0.01, 0.1, 1 and 10 µg/mL of AgGom or HiGom and the control was salt de-chlorinated tap water (SDTW). During the duration of the experiments, the mortality of the embryos was recorded at 24, 48 and 120 h post treatment.

### Zebrafish embryo xenograft assays

Zebrafish embryos were collected and incubated at 28.5 °C during the first 48 h hpf. At 48 hpf the embryos were anaesthetized with 0.003% tricaine (Sigma). MM96L human melanoma cells were cultured at 37 °C and 5% CO_2_ until they reached ~70% confluence. MM96L cells were then trypsinized and one million cells were harvested, labeled with Dil lipophilic dye and concentrated in 10 µL of phosphate-buffered saline (PBS) containing 2% polyvinyl-pyrrolidone 40 (PVP40) to avoid cell aggregation. Borosilicate glass capillary needles (1 mm O.D. × 0.75 mm I.D.; World Precision Instruments) were used to inject 200–300 cells into the circulation of the embryos using a micromanipulator and a IM-31 Electric Microinjector (Narishige) with an output pressure of 34 kPa and 30 ms injection time. After the injection, embryos were incubated for three days post-injection (hpi) at 34 °C in 30-mL Petri dishes containing SDTW.

### Zebrafish embryo image analysis

Imaging of embryos was performed with an AZ-100 Nikon fluorescence stereomicroscope at 24 hpi and 96 hpi, which allowing live tracking of the labeled proliferating MM96L cells inside the zebrafish embryo. Analysis of the images taken at two different times (days 1 and 3) to monitor progression of the injected cells, was performed using Quantifish Software^54^. This software quantifies the fluorescence of each image above a manually established threshold, yielding the integrated density of each image as an output. This measurement is the result of multiplying the number of positive pixels by the medium intensity of the fluorescence per each image. Integrated density was then processed to obtain a proliferation ratio and to compare the difference and normalized to the control conditions of the experiment.

### Statistical analysis

Statistical analysis was performed using Graphpad Software (GraphPad Prism version 7.00 for Windows, GraphPad Software, La Jolla California USA). All data represent a minimum of three independent experiments and are expressed as the mean ± standard error of the mean (SEM). Statistical analyses were conducted using either a Student’s *t-*test or ANOVA for comparison between groups and control. Statistical significance was considered at *P < 0.05, **P < 0.01, ***P < 0.001 or ****P < 0.0001. When populations within groups were not normally distributed, a non-parametric Mann-Whitney test was used. For mouse *in vivo* studies, the permutation test was used to compare tumor progression between HiGom-treated and control groups. In zebrafish embryo xenograft assays, the outliers were identified using ‘Identify Outliers’ test followed by a *t*-test analysis. *P < 0.05 was considered statistically significant for all experiments.

### Data availability

All data supporting this work are available within this article and the supplementary section, or available from the authors upon request.

### Data deposition

Metadata and annotated nucleotide sequences generated and utilised in this work were deposited on the European nucleotide archive (ENA) under project accession number ERA298588. HiGom peptide can be found under accession HACE01000099.

## Electronic supplementary material

Supplementary File
