# Supplementary material for: Gomesin inhibits melanoma growth by manipulating key signaling cascades that control cell death and proliferation
Source: Sci Rep. 2018 Aug 1;8:11519. doi: 10.1038/s41598-018-29826-4 (PMC6070509; doi:10.1038/s41598-018-29826-4)
Supplement: Supplementary file 1 — Supplementary File [file 41598_2018_29826_MOESM1_ESM.docx]

**Supplementary Information**

**Gomesin inhibits melanoma growth by manipulating key signaling cascades that control cell death and proliferation**

**Maria P. Ikonomopoulou, Manuel A. Fernandez-Rojo, Sandy S. Pineda, Pablo Cabezas Sainz, Brit Winnen, Rodrigo A.V. Morales, Andreas Brust, Laura Elena Sánchez, Paul Alewood, Grant A. Ramm, John J. Miles and Glenn F. King**

**Supplementary Figure 1**. Hemolytic activity of AgGom and HiGom compared to melittin.

**Supplementary Figure 2.** Gomesin peptides dramatically reduce the viability of melanoma BRAF-mutated cells. Viability of MM96L, A2058, HTT144, JA, SKMEL28 and A02 cells treated with 50 µg/mL of **(a)** AgGom or **(b)** HiGom for 48 h*.* Data are mean ± SEM. Experiments were performed in triplicate and are the result of three independent experiments.


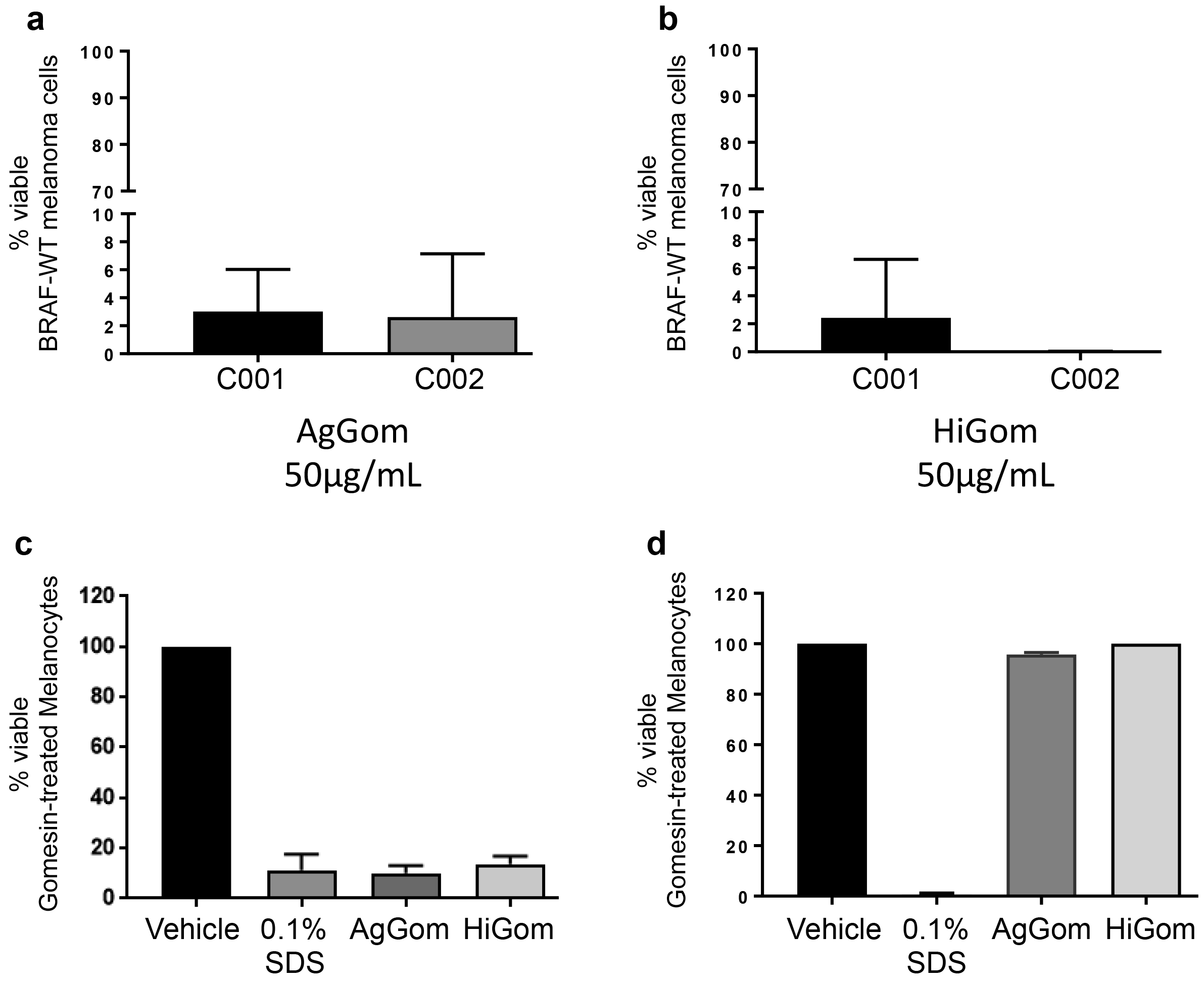


**Supplementary Figure 3. The effect of gomesin peptides on the viability of BRAF-wild type cells and melanocytes**. (a) AgGom or (b) HiGom-treated BRAF-wild type cells at 50 µg/mL and gomesin-treated melanocytes at (c) 50 µg/mL and (d) 12.5 µg/mL for 48 h, compared to cells treated with 0.1% SDS and untreated cells.


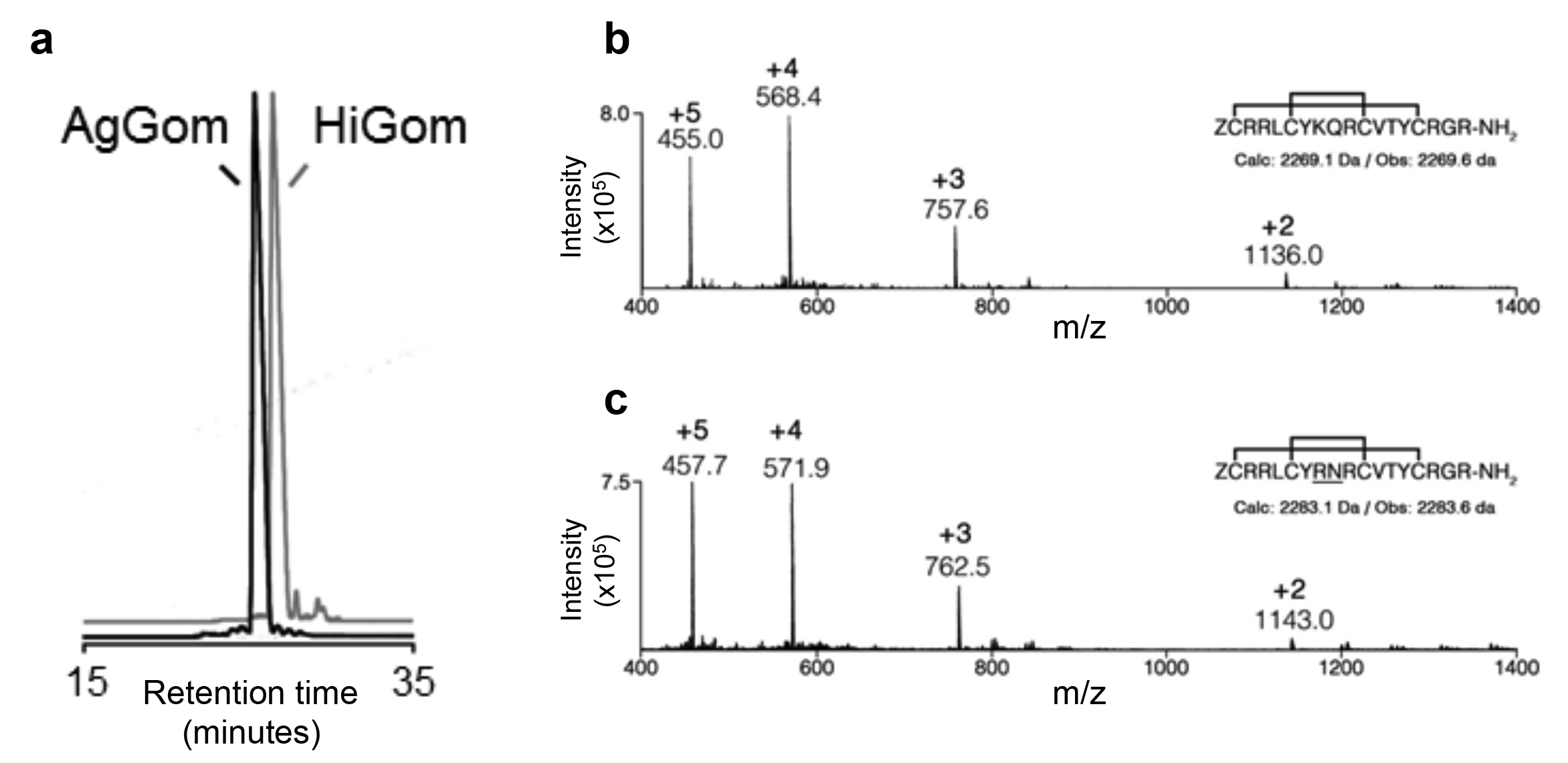


**Supplementary Figure 4**. **(a)** RP-HPLC chromatograms comparing the retention times of synthetic folded HiGom and AgGom peptides. Peptides were eluted from a Vydac C18 column (4.6 x 250 mm) using a linear acetonitrile gradient (Buffer: acetonitrile/water/TFA, 90:10:0.043) over 40 min at a flow rate 1 mL/min. MS spectra of synthetic (**b**) AgGom and **(c)** HiGom.


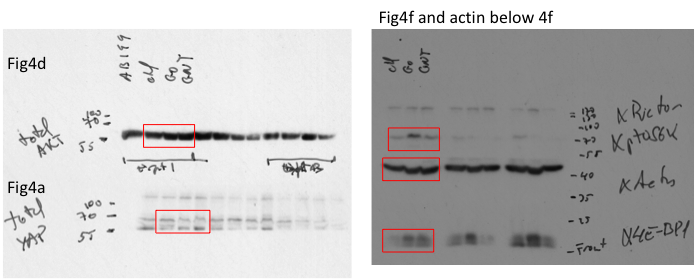


**Supplementary Figure 5**. Full-length gels of Figure 4d, 4f. Note: Go=AgGom, GNT= HiGom and AB199= other compound used for another study.


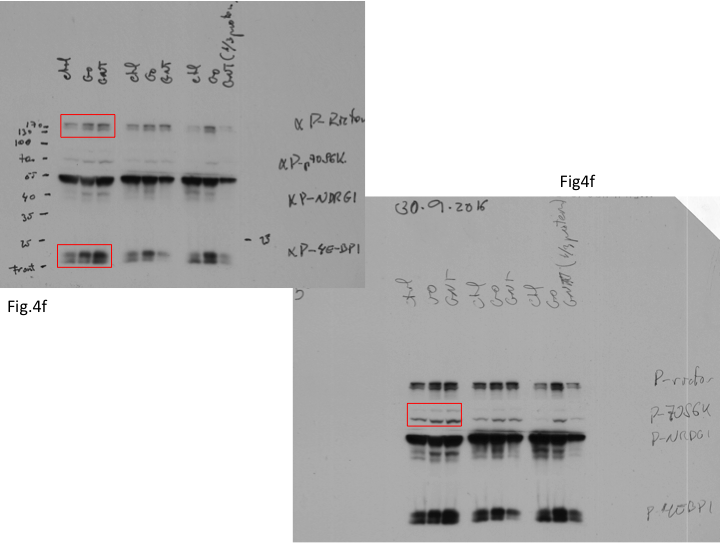


**Supplementary Figure 6**. Full-length gels of Figure 4f. Note: Go=AgGom, GNT= HiGom and Ctrl= vehicle treated-cells.


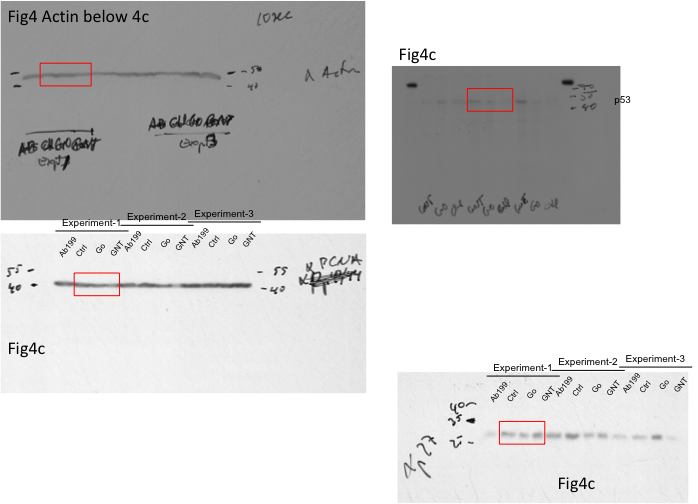


**Supplementary Figure 7**. Full-length gels of Figure 4c. Note: Go=AgGom, GNT= HiGom, Ctrl= vehicle treated-cells and AB199= other compound used for another study.


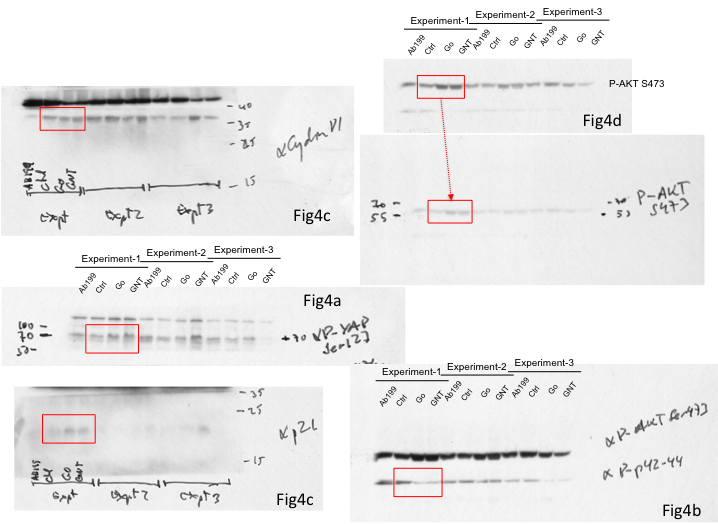


**Supplementary Figure 8**. Full-length gels of Figure 4a-d. Note: Go=AgGom, GNT= HiGom, Ctrl= vehicle treated-cells and AB199= other compound used for another study.


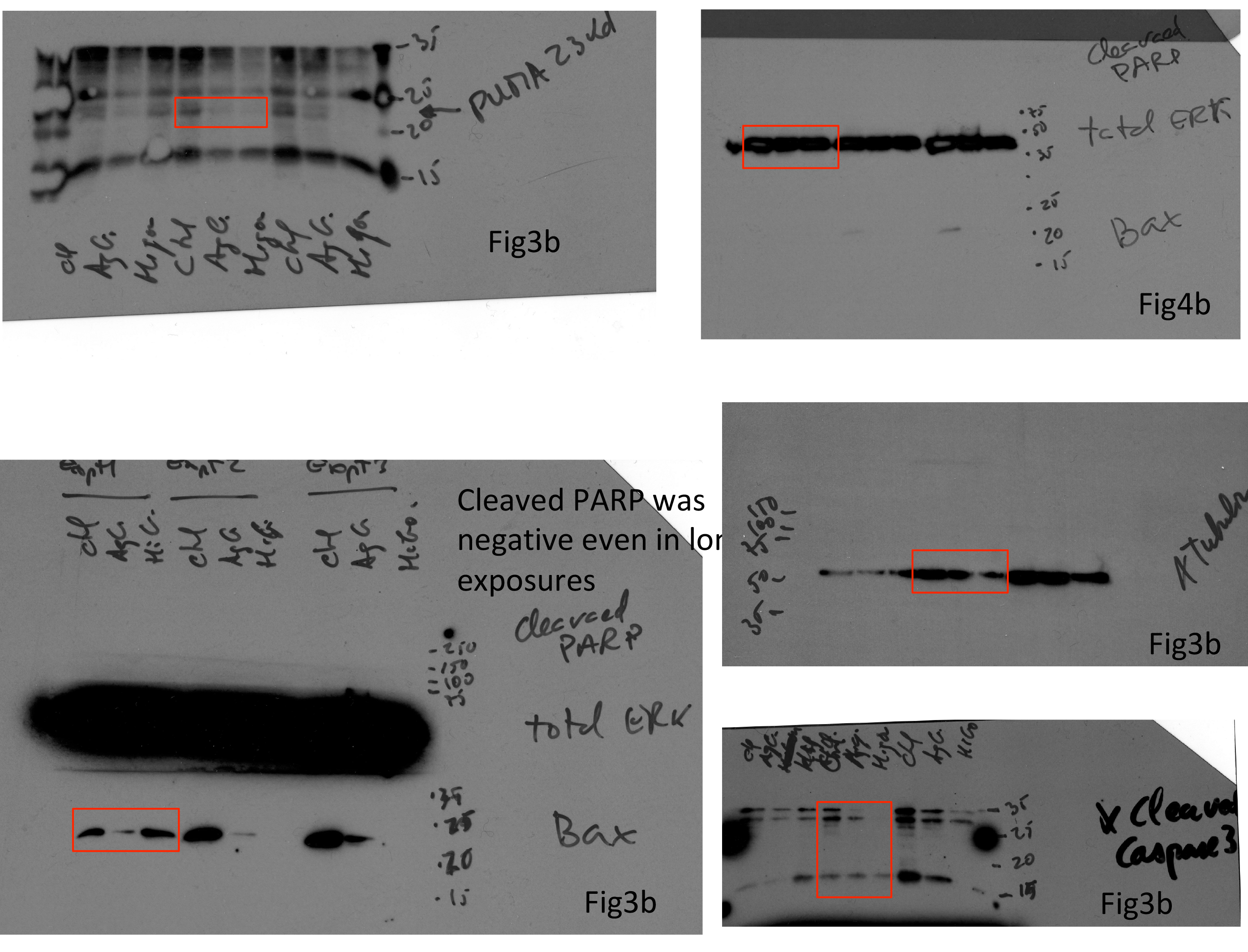


**Supplementary Figure 9**. Full-length gels of Figures 3c & 4b. Note: Go=AgGom, GNT= HiGom, Ctrl= vehicle treated-cells.


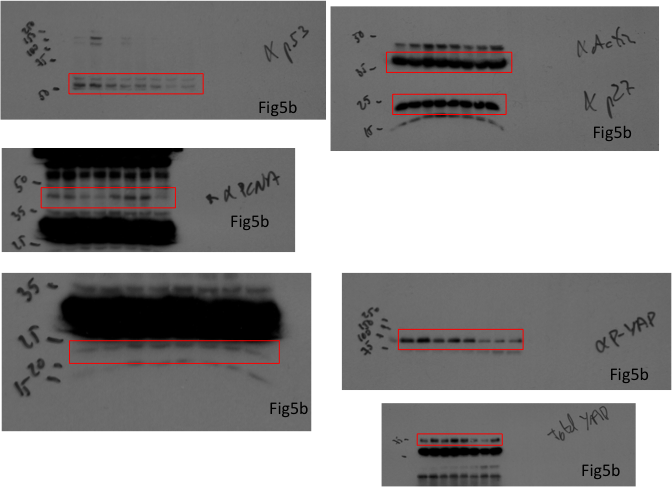


**Supplementary Figure 10**. Full-length gels of Figure 5b for analyzing the HiGom-treated and vehicle-treated MM96L xenograft tumors.

**Supplementary Table 1:** Toxicity assessment of zebrafish embryos exposed to AgGom or HiGom for 24 h, 48 h or 120 h. Three independent experiments were performed with 12 replicates per group. Bold indicates the optimal chosen dose for xenograft experiments.

| **REPLICATE 1** | **AgGom** | **HiGom** | **AgGom** | **HiGom** | **AgGom** | **HiGom** |
| --- | --- | --- | --- | --- | --- | --- |
| **Control (0 μg/mL)** | 0/12 | 0/12 | 0/12 | 0/12 | 0/12 | 0/12 |
| **0.01 μg/mL** | 0/12 | 0/12 | 0/12 | 1/12 | 0/12 | 3/12 |
| **0.1 μg/mL** | **0/12** | **0/12** | **1/12** | **1/12** | **2/12** | **5/12** |
| **1 μg/mL** | 3/12 | 6/12 | 3/12 | 6/12 | 5/12 | 6/12 |
| **10 μg/mL** | 12/12 | 12/12 | 12/12 | 12/12 | 12/12 | 12/12 |
| **REPLICATE 2** | **AgGom** | **HiGom** | **AgGom** | **HiGom** | **AgGom** | **HiGom** |
| **Control (0 μg/mL)** | 0/12 | 0/12 | 0/12 | 0/12 | 1/12 | 1/12 |
| **0.01 μg/mL** | 0/12 | 0/12 | 0/12 | 0/12 | 1/12 | 0/12 |
| **0.1 μg/mL** | **0/12** | **0/12** | **0/12** | **0/12** | **0/12** | **0/12** |
| **1 μg/mL** | 4/12 | 10/12 | 4/12 | 10/12 | 6/12 | 10/12 |
| **10 μg/mL** | 12/12 | 12/12 | 12/12 | 12/12 | 12/12 | 12/12 |
| **REPLICATE 3** | **AgGom** | **HiGom** | **AgGom** | **HiGom** | **AgGom** | **HiGom** |
| **Control (0 μg/mL)** | 0/12 | 0/12 | 0/12 | 0/12 | 1/12 | 1/12 |
| **0.01 μg/mL** | 0/12 | 0/12 | 0/12 | 0/12 | 2/12 | 1/12 |
| **0.1 μg/mL** | **0/12** | **0/12** | **0/12** | **0/12** | **3/12** | **1/12** |
| **1 μg/mL** | 4/12 | 7/12 | 4/12 | 7/12 | 5/12 | 7/12 |
| **10 μg/mL** | 12/12 | 12/12 | 12/12 | 12/12 | 12/12 | 12/12 |
